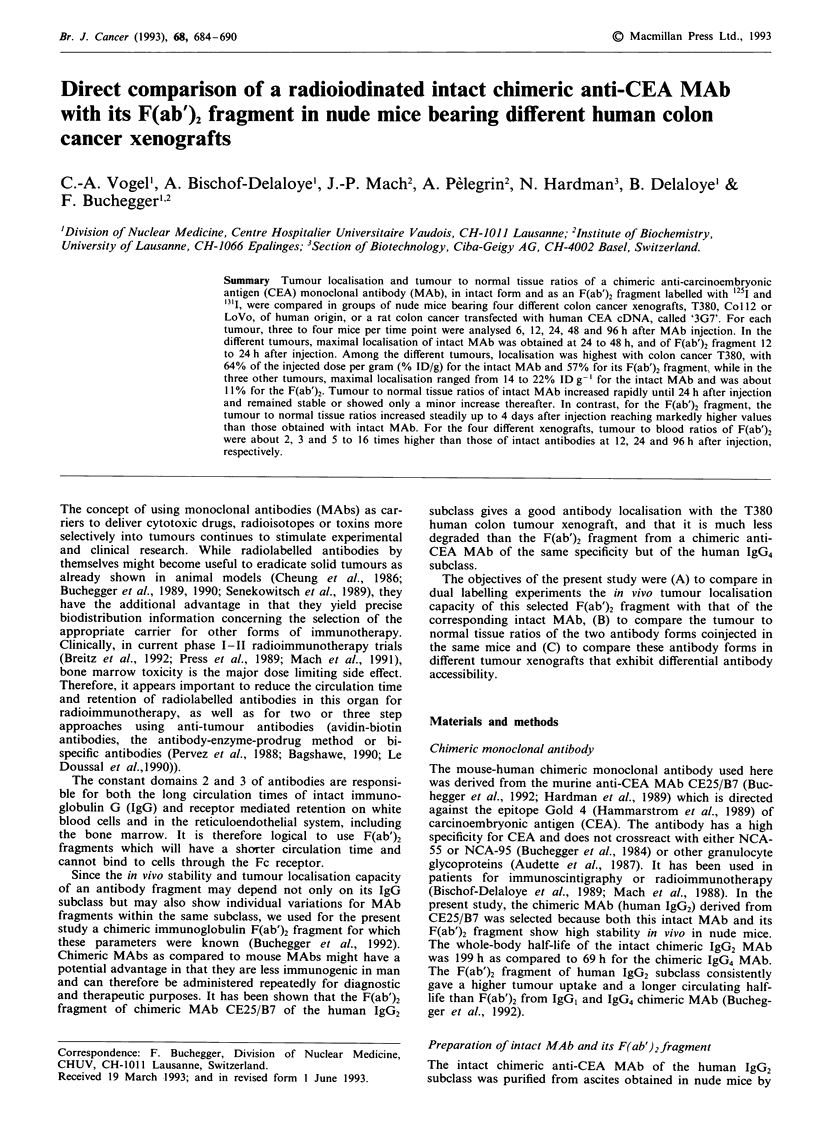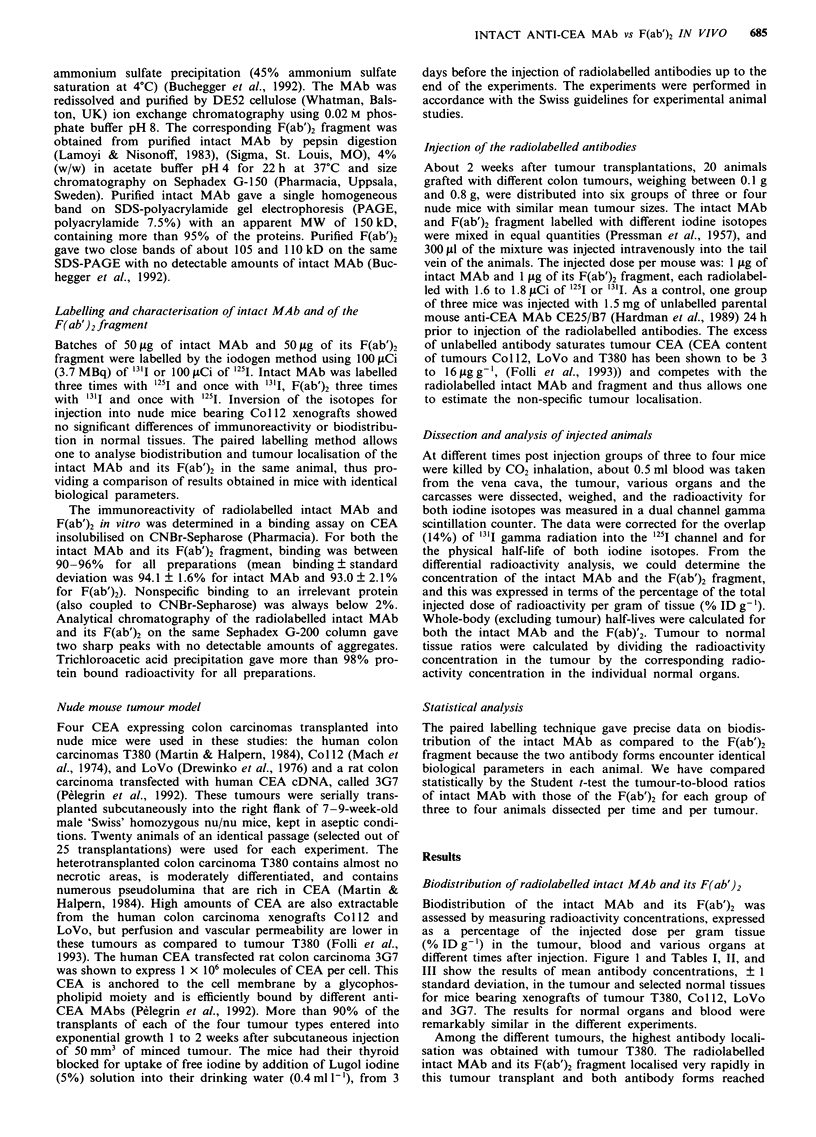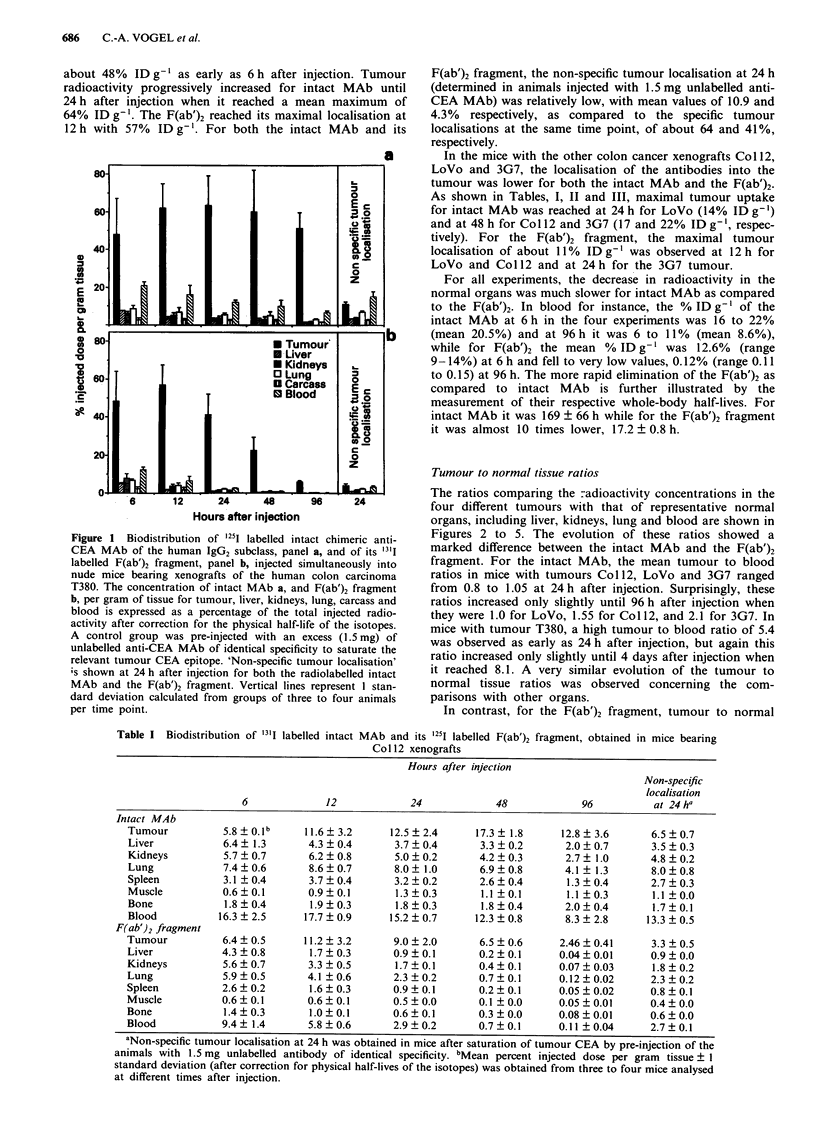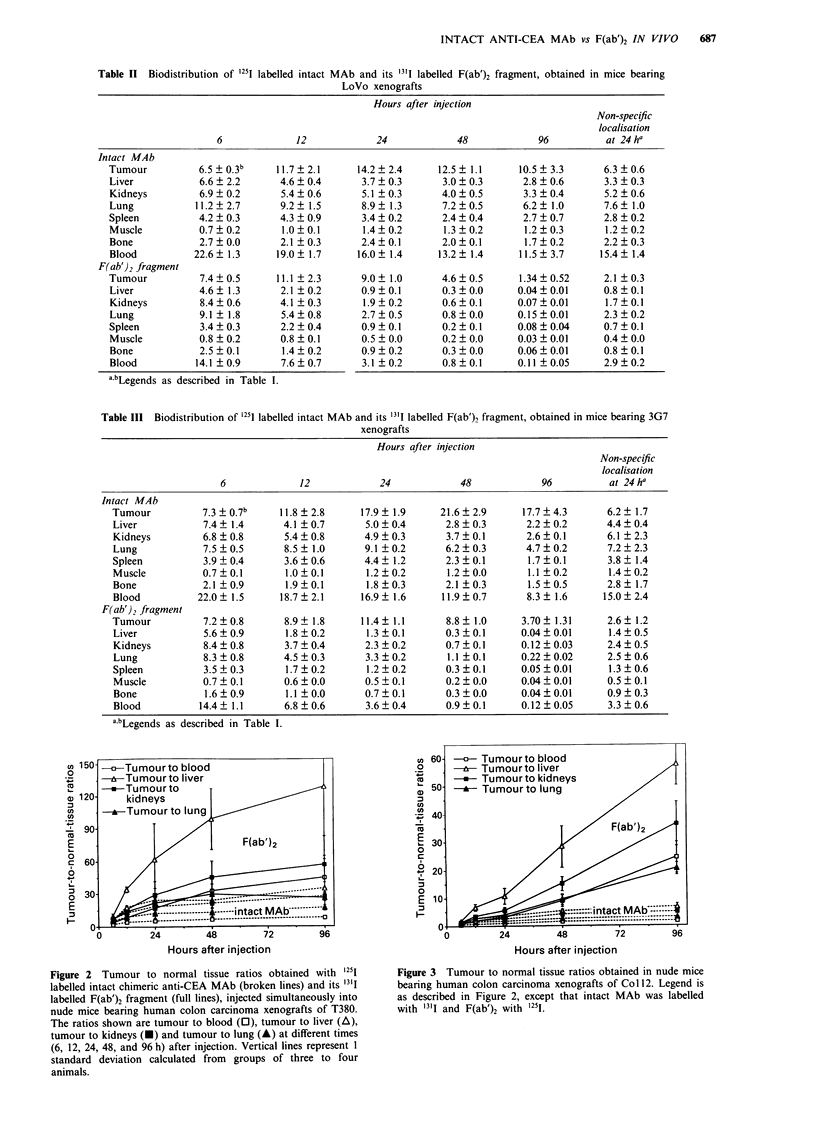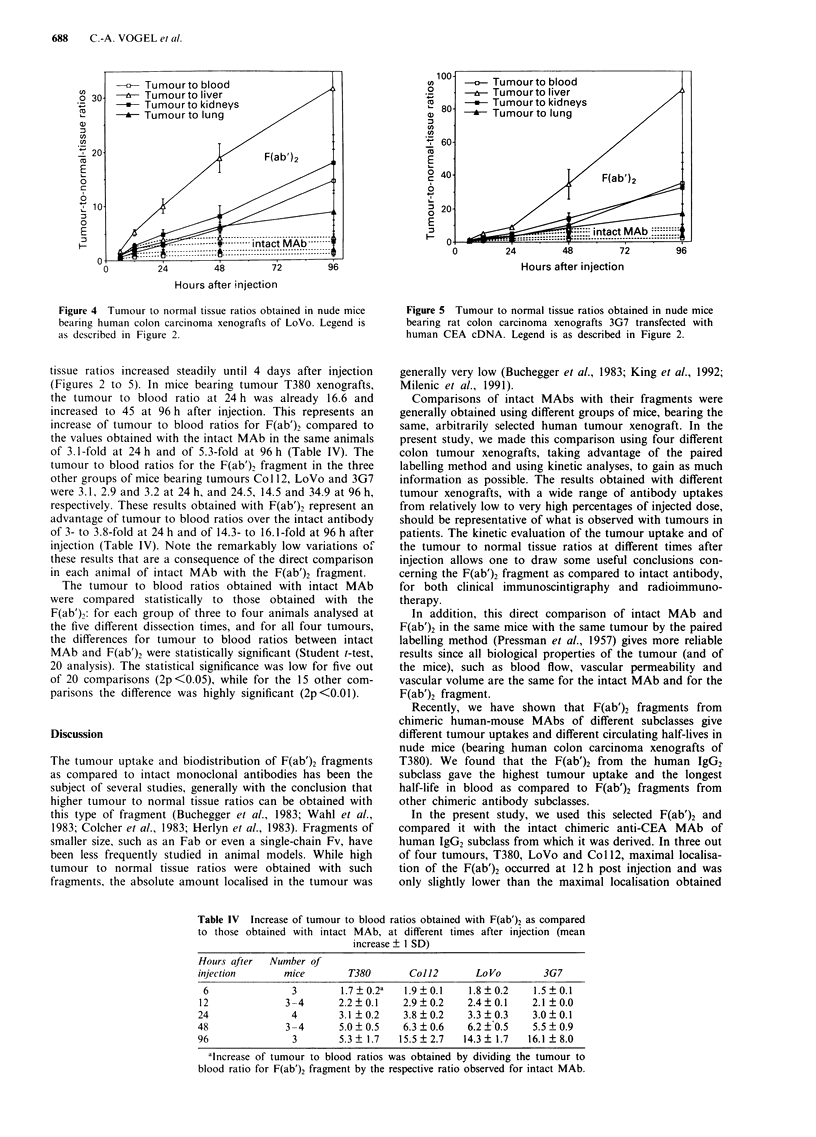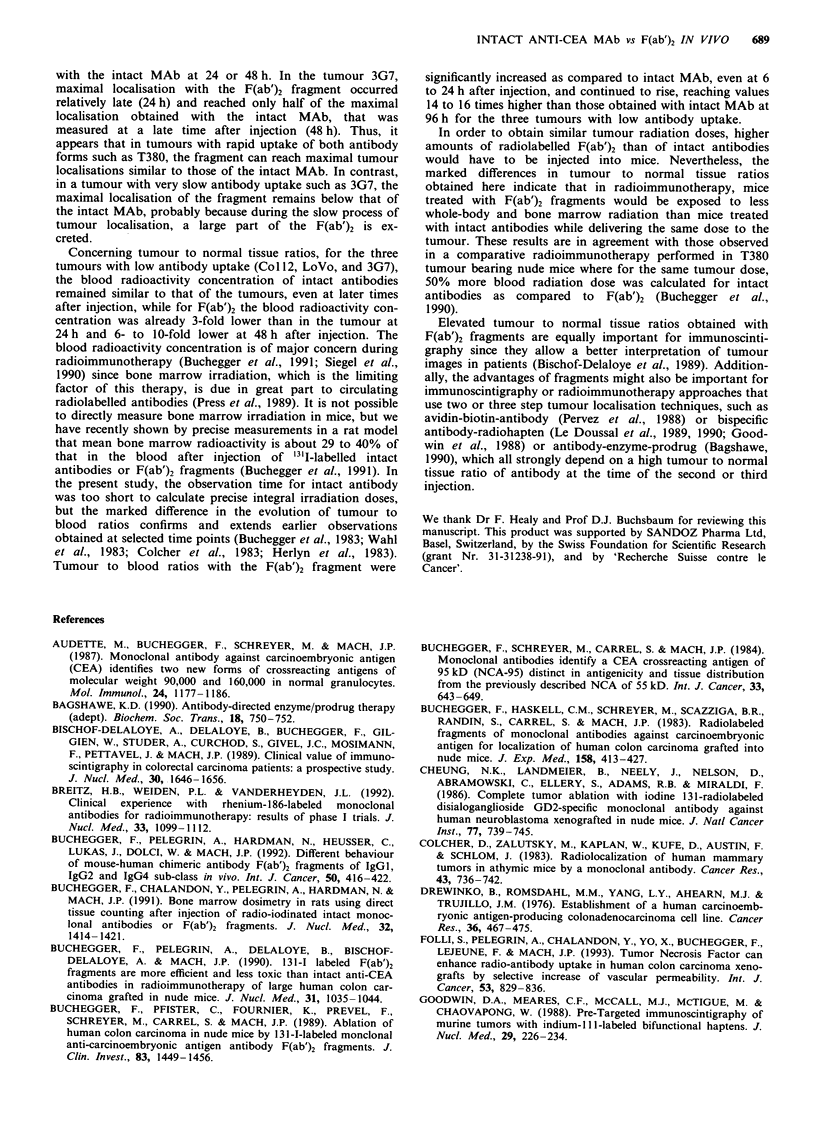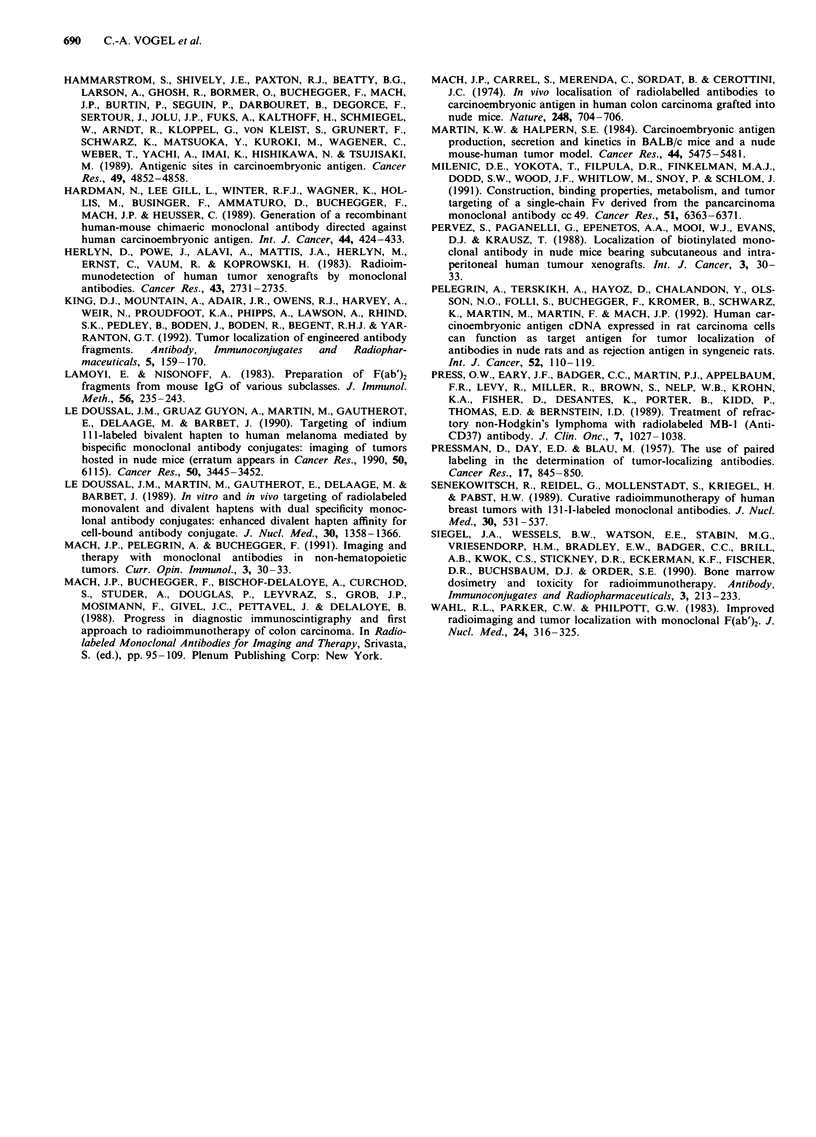# Direct comparison of a radioiodinated intact chimeric anti-CEA MAb with its F(ab')2 fragment in nude mice bearing different human colon cancer xenografts.

**DOI:** 10.1038/bjc.1993.410

**Published:** 1993-10

**Authors:** C. A. Vogel, A. Bischof-Delaloye, J. P. Mach, A. Pèlegrin, N. Hardman, B. Delaloye, F. Buchegger

**Affiliations:** Division of Nuclear Medicine, Centre Hospitalier Universitaier Vaudois, Lausanne, Switzerland.

## Abstract

Tumour localisation and tumour to normal tissue ratios of a chimeric anti-carcinoembryonic antigen (CEA) monoclonal antibody (MAb), in intact form and as an F(ab')2 fragment labelled with 125I and 131I, were compared in groups of nude mice bearing four different colon cancer xenografts, T380, Co112 or LoVo, of human origin, or a rat colon cancer transfected with human CEA cDNA, called '3G7'. For each tumour, three to four mice per time point were analysed 6, 12, 24, 48 and 96 h after MAb injection. In the different tumours, maximal localisation of intact MAb was obtained at 24 to 48 h, and of F(ab')2 fragment 12 to 24 h after injection. Among the different tumours, localisation was highest with colon cancer T380, with 64% of the injected dose per gram (% ID/g) for the intact MAb and 57% for its F(ab')2 fragment, while in the three other tumours, maximal localisation ranged from 14 to 22% ID g-1 for the intact MAb and was about 11% for the F(ab')2. Tumour to normal tissue ratios of intact MAb increased rapidly until 24 h after injection and remained stable or showed only a minor increase thereafter. In contrast, for the F(ab')2 fragment, the tumour to normal tissue ratios increased steadily up to 4 days after injection reaching markedly higher values than those obtained with intact MAb. For the four different xenografts, tumour to blood ratios of F(ab')2 were about 2, 3 and 5 to 16 times higher than those of intact antibodies at 12, 24 and 96 h after injection, respectively.


Br. J. Cancer (1993), 68, 684–690 © Macmillan Press Ltd., 1993

### Direct comparison of a radioiodinated intact chimeric anti-CEA MAb with its F(ab′)$_2$ fragment in nude mice bearing different human colon cancer xenografts

C.-A. Vogel[1], A. Bischof-Delaloye[1], J.-P. Mach[2], A. Pèlegrin[2], N. Hardman[3], B. Delaloye[1] & F. Buchegger[1,2]

[1]*Division of Nuclear Medicine, Centre Hospitalier Universitaire Vaudois, CH-1011 Lausanne;* [2]*Institute of Biochemistry, University of Lausanne, CH-1066 Epalinges;* [3]*Section of Biotechnology, Ciba-Geigy AG, CH-4002 Basel, Switzerland.*

**Summary** Tumour localisation and tumour to normal tissue ratios of a chimeric anti-carcinoembryonic antigen (CEA) monoclonal antibody (MAb), in intact form and as an F(ab′)$_2$ fragment labelled with $^{125}$I and $^{131}$I, were compared in groups of nude mice bearing four different colon cancer xenografts, T380, Co112 or LoVo, of human origin, or a rat colon cancer transfected with human CEA cDNA, called '3G7'. For each tumour, three to four mice per time point were analysed 6, 12, 24, 48 and 96 h after MAb injection. In the different tumours, maximal localisation of intact MAb was obtained at 24 to 48 h, and of F(ab′)$_2$ fragment 12 to 24 h after injection. Among the different tumours, localisation was highest with colon cancer T380, with 64% of the injected dose per gram (% ID/g) for the intact MAb and 57% for its F(ab′)$_2$ fragment, while in the three other tumours, maximal localisation ranged from 14 to 22% ID g$^{-1}$ for the intact MAb and was about 11% for the F(ab′)$_2$. Tumour to normal tissue ratios of intact MAb increased rapidly until 24 h after injection and remained stable or showed only a minor increase thereafter. In contrast, for the F(ab′)$_2$ fragment, the tumour to normal tissue ratios increased steadily up to 4 days after injection reaching markedly higher values than those obtained with intact MAb. For the four different xenografts, tumour to blood ratios of F(ab′)$_2$ were about 2, 3 and 5 to 16 times higher than those of intact antibodies at 12, 24 and 96 h after injection, respectively.

The concept of using monoclonal antibodies (MAbs) as carriers to deliver cytotoxic drugs, radioisotopes or toxins more selectively into tumours continues to stimulate experimental and clinical research. While radiolabelled antibodies by themselves might become useful to eradicate solid tumours as already shown in animal models (Cheung *et al.*, 1986; Buchegger *et al.*, 1989, 1990; Senekowitsch *et al.*, 1989), they have the additional advantage in that they yield precise biodistribution information concerning the selection of the appropriate carrier for other forms of immunotherapy. Clinically, in current phase I–II radioimmunotherapy trials (Breitz *et al.*, 1992; Press *et al.*, 1989; Mach *et al.*, 1991), bone marrow toxicity is the major dose limiting side effect. Therefore, it appears important to reduce the circulation time and retention of radiolabelled antibodies in this organ for radioimmunotherapy, as well as for two or three step approaches using anti-tumour antibodies (avidin-biotin antibodies, the antibody-enzyme-prodrug method or bi-specific antibodies (Pervez *et al.*, 1988; Bagshawe, 1990; Le Doussal *et al.*,1990)).

The constant domains 2 and 3 of antibodies are responsible for both the long circulation times of intact immunoglobulin G (IgG) and receptor mediated retention on white blood cells and in the reticuloendothelial system, including the bone marrow. It is therefore logical to use F(ab′)$_2$ fragments which will have a shorter circulation time and cannot bind to cells through the Fc receptor.

Since the *in vivo* stability and tumour localisation capacity of an antibody fragment may depend not only on its IgG subclass but may also show individual variations for MAb fragments within the same subclass, we used for the present study a chimeric immunoglobulin F(ab′)$_2$ fragment for which these parameters were known (Buchegger *et al.*, 1992). Chimeric MAbs as compared to mouse MAbs might have a potential advantage in that they are less immunogenic in man and can therefore be administered repeatedly for diagnostic and therapeutic purposes. It has been shown that the F(ab′)$_2$ fragment of chimeric MAb CE25/B7 of the human IgG$_2$ subclass gives a good antibody localisation with the T380 human colon tumour xenograft, and that it is much less degraded than the F(ab′)$_2$ fragment from a chimeric anti-CEA MAb of the same specificity but of the human IgG$_4$ subclass.

The objectives of the present study were (A) to compare in dual labelling experiments the *in vivo* tumour localisation capacity of this selected F(ab′)$_2$ fragment with that of the corresponding intact MAb, (B) to compare the tumour to normal tissue ratios of the two antibody forms coinjected in the same mice and (C) to compare these antibody forms in different tumour xenografts that exhibit differential antibody accessibility.

Correspondence: F. Buchegger, Division of Nuclear Medicine, CHUV, CH-1011 Lausanne, Switzerland.
Received 19 March 1993; and in revised form 1 June 1993.

### Materials and methods

### *Chimeric monoclonal antibody*

The mouse-human chimeric monoclonal antibody used here was derived from the murine anti-CEA MAb CE25/B7 (Buchegger *et al.*, 1992; Hardman *et al.*, 1989) which is directed against the epitope Gold 4 (Hammarstrom *et al.*, 1989) of carcinoembryonic antigen (CEA). The antibody has a high specificity for CEA and does not crossreact with either NCA-55 or NCA-95 (Buchegger *et al.*, 1984) or other granulocyte glycoproteins (Audette *et al.*, 1987). It has been used in patients for immunoscintigraphy or radioimmunotherapy (Bischof-Delaloye *et al.*, 1989; Mach *et al.*, 1988). In the present study, the chimeric MAb (human IgG$_2$) derived from CE25/B7 was selected because both this intact MAb and its F(ab′)$_2$ fragment show high stability *in vivo* in nude mice. The whole-body half-life of the intact chimeric IgG$_2$ MAb was 199 h as compared to 69 h for the chimeric IgG$_4$ MAb. The F(ab′)$_2$ fragment of human IgG$_2$ subclass consistently gave a higher tumour uptake and a longer circulating half-life than F(ab′)$_2$ from IgG$_1$ and IgG$_4$ chimeric MAb (Buchegger *et al.*, 1992).

### *Preparation of intact MAb and its F(ab′)$_2$ fragment*

The intact chimeric anti-CEA MAb of the human IgG$_2$ subclass was purified from ascites obtained in nude mice by ammonium sulfate precipitation (45% ammonium sulfate saturation at 4°C) (Buchegger *et al.*, 1992). The MAb was redissolved and purified by DE52 cellulose (Whatman, Balston, UK) ion exchange chromatography using 0.02 M phosphate buffer pH 8. The corresponding F(ab')$_2$ fragment was obtained from purified intact MAb by pepsin digestion (Lamoyi & Nisonoff, 1983), (Sigma, St. Louis, MO), 4% (w/w) in acetate buffer pH 4 for 22 h at 37°C and size chromatography on Sephadex G-150 (Pharmacia, Uppsala, Sweden). Purified intact MAb gave a single homogeneous band on SDS-polyacrylamide gel electrophoresis (PAGE, polyacrylamide 7.5%) with an apparent MW of 150 kD, containing more than 95% of the proteins. Purified F(ab')$_2$ gave two close bands of about 105 and 110 kD on the same SDS-PAGE with no detectable amounts of intact MAb (Buchegger *et al.*, 1992).

*Labelling and characterisation of intact MAb and of the F(ab')$_2$ fragment*

Batches of 50 μg of intact MAb and 50 μg of its F(ab')$_2$ fragment were labelled by the iodogen method using 100 μCi (3.7 MBq) of $^{131}$I or 100 μCi of $^{125}$I. Intact MAb was labelled three times with $^{125}$I and once with $^{131}$I, F(ab')$_2$ three times with $^{131}$I and once with $^{125}$I. Inversion of the isotopes for injection into nude mice bearing Co112 xenografts showed no significant differences of immunoreactivity or biodistribution in normal tissues. The paired labelling method allows one to analyse biodistribution and tumour localisation of the intact MAb and its F(ab')$_2$ in the same animal, thus providing a comparison of results obtained in mice with identical biological parameters.

The immunoreactivity of radiolabelled intact MAb and F(ab')$_2$ *in vitro* was determined in a binding assay on CEA insolubilised on CNBr-Sepharose (Pharmacia). For both the intact MAb and its F(ab')$_2$ fragment, binding was between 90–96% for all preparations (mean binding ± standard deviation was 94.1 ± 1.6% for intact MAb and 93.0 ± 2.1% for F(ab')$_2$). Nonspecific binding to an irrelevant protein (also coupled to CNBr-Sepharose) was always below 2%. Analytical chromatography of the radiolabelled intact MAb and its F(ab')$_2$ on the same Sephadex G-200 column gave two sharp peaks with no detectable amounts of aggregates. Trichloroacetic acid precipitation gave more than 98% protein bound radioactivity for all preparations.

*Nude mouse tumour model*

Four CEA expressing colon carcinomas transplanted into nude mice were used in these studies: the human colon carcinomas T380 (Martin & Halpern, 1984), Co112 (Mach *et al.*, 1974), and LoVo (Drewinko *et al.*, 1976) and a rat colon carcinoma transfected with human CEA cDNA, called 3G7 (Pèlegrin *et al.*, 1992). These tumours were serially transplanted subcutaneously into the right flank of 7–9-week-old male 'Swiss' homozygous nu/nu mice, kept in aseptic conditions. Twenty animals of an identical passage (selected out of 25 transplantations) were used for each experiment. The heterotransplanted colon carcinoma T380 contains almost no necrotic areas, is moderately differentiated, and contains numerous pseudolumina that are rich in CEA (Martin & Halpern, 1984). High amounts of CEA are also extractable from the human colon carcinoma xenografts Co112 and LoVo, but perfusion and vascular permeability are lower in these tumours as compared to tumour T380 (Folli *et al.*, 1993). The human CEA transfected rat colon carcinoma 3G7 was shown to express 1 × 10$^6$ molecules of CEA per cell. This CEA is anchored to the cell membrane by a glycophospholipid moiety and is efficiently bound by different anti-CEA MAbs (Pèlegrin *et al.*, 1992). More than 90% of the transplants of each of the four tumour types entered into exponential growth 1 to 2 weeks after subcutaneous injection of 50 mm$^3$ of minced tumour. The mice had their thyroid blocked for uptake of free iodine by addition of Lugol iodine (5%) solution into their drinking water (0.4 ml l$^{-1}$), from 3 days before the injection of radiolabelled antibodies up to the end of the experiments. The experiments were performed in accordance with the Swiss guidelines for experimental animal studies.

*Injection of the radiolabelled antibodies*

About 2 weeks after tumour transplantations, 20 animals grafted with different colon tumours, weighing between 0.1 g and 0.8 g, were distributed into six groups of three or four nude mice with similar mean tumour sizes. The intact MAb and F(ab')$_2$ fragment labelled with different iodine isotopes were mixed in equal quantities (Pressman *et al.*, 1957), and 300 μl of the mixture was injected intravenously into the tail vein of the animals. The injected dose per mouse was: 1 μg of intact MAb and 1 μg of its F(ab')$_2$ fragment, each radiolabelled with 1.6 to 1.8 μCi of $^{125}$I or $^{131}$I. As a control, one group of three mice was injected with 1.5 mg of unlabelled parental mouse anti-CEA MAb CE25/B7 (Hardman *et al.*, 1989) 24 h prior to injection of the radiolabelled antibodies. The excess of unlabelled antibody saturates tumour CEA (CEA content of tumours Co112, LoVo and T380 has been shown to be 3 to 16 μg g$^{-1}$, (Folli *et al.*, 1993)) and competes with the radiolabelled intact MAb and fragment and thus allows one to estimate the non-specific tumour localisation.

*Dissection and analysis of injected animals*

At different times post injection groups of three to four mice were killed by $CO_2$ inhalation, about 0.5 ml blood was taken from the vena cava, the tumour, various organs and the carcasses were dissected, weighed, and the radioactivity for both iodine isotopes was measured in a dual channel gamma scintillation counter. The data were corrected for the overlap (14%) of $^{131}$I gamma radiation into the $^{125}$I channel and for the physical half-life of both iodine isotopes. From the differential radioactivity analysis, we could determine the concentration of the intact MAb and the F(ab')$_2$ fragment, and this was expressed in terms of the percentage of the total injected dose of radioactivity per gram of tissue (% ID g$^{-1}$). Whole-body (excluding tumour) half-lives were calculated for both the intact MAb and the F(ab)'$_2$. Tumour to normal tissue ratios were calculated by dividing the radioactivity concentration in the tumour by the corresponding radioactivity concentration in the individual normal organs.

*Statistical analysis*

The paired labelling technique gave precise data on biodistribution of the intact MAb as compared to the F(ab')$_2$ fragment because the two antibody forms encounter identical biological parameters in each animal. We have compared statistically by the Student *t*-test the tumour-to-blood ratios of intact MAb with those of the F(ab')$_2$ for each group of three to four animals dissected per time and per tumour.

### Results

*Biodistribution of radiolabelled intact MAb and its F(ab')$_2$*

Biodistribution of the intact MAb and its F(ab')$_2$ was assessed by measuring radioactivity concentrations, expressed as a percentage of the injected dose per gram tissue (% ID g$^{-1}$) in the tumour, blood and various organs at different times after injection. Figure 1 and Tables I, II, and III show the results of mean antibody concentrations, ± 1 standard deviation, in the tumour and selected normal tissues for mice bearing xenografts of tumour T380, Co112, LoVo and 3G7. The results for normal organs and blood were remarkably similar in the different experiments.

Among the different tumours, the highest antibody localisation was obtained with tumour T380. The radiolabelled intact MAb and its F(ab')$_2$ fragment localised very rapidly in this tumour transplant and both antibody forms reached about 48% ID $g^{-1}$ as early as 6 h after injection. Tumour radioactivity progressively increased for intact MAb until 24 h after injection when it reached a mean maximum of 64% ID $g^{-1}$. The $F(ab')_2$ reached its maximal localisation at 12 h with 57% ID $g^{-1}$. For both the intact MAb and its $F(ab')_2$ fragment, the non-specific tumour localisation at 24 h (determined in animals injected with 1.5 mg unlabelled anti-CEA MAb) was relatively low, with mean values of 10.9 and 4.3% respectively, as compared to the specific tumour localisations at the same time point, of about 64 and 41%, respectively.

**Figure 1** Biodistribution of $^{125}I$ labelled intact chimeric anti-CEA MAb of the human $IgG_2$ subclass, panel **a**, and of its $^{131}I$ labelled $F(ab')_2$ fragment, panel **b**, injected simultaneously into nude mice bearing xenografts of the human colon carcinoma T380. The concentration of intact MAb **a**, and $F(ab')_2$ fragment **b**, per gram of tissue for tumour, liver, kidneys, lung, carcass and blood is expressed as a percentage of the total injected radioactivity after correction for the physical half-life of the isotopes. A control group was pre-injected with an excess (1.5 mg) of unlabelled anti-CEA MAb of identical specificity to saturate the relevant tumour CEA epitope. 'Non-specific tumour localisation' is shown at 24 h after injection for both the radiolabelled intact MAb and the $F(ab')_2$ fragment. Vertical lines represent 1 standard deviation calculated from groups of three to four animals per time point.

In the mice with the other colon cancer xenografts Co112, LoVo and 3G7, the localisation of the antibodies into the tumour was lower for both the intact MAb and the $F(ab')_2$. As shown in Tables, I, II and III, maximal tumour uptake for intact MAb was reached at 24 h for LoVo (14% ID $g^{-1}$) and at 48 h for Co112 and 3G7 (17 and 22% ID $g^{-1}$, respectively). For the $F(ab')_2$ fragment, the maximal tumour localisation of about 11% ID $g^{-1}$ was observed at 12 h for LoVo and Co112 and at 24 h for the 3G7 tumour.

For all experiments, the decrease in radioactivity in the normal organs was much slower for intact MAb as compared to the $F(ab')_2$. In blood for instance, the % ID $g^{-1}$ of the intact MAb at 6 h in the four experiments was 16 to 22% (mean 20.5%) and at 96 h it was 6 to 11% (mean 8.6%), while for $F(ab')_2$ the mean % ID $g^{-1}$ was 12.6% (range 9–14%) at 6 h and fell to very low values, 0.12% (range 0.11 to 0.15) at 96 h. The more rapid elimination of the $F(ab')_2$ as compared to intact MAb is further illustrated by the measurement of their respective whole-body half-lives. For intact MAb it was 169 ± 66 h while for the $F(ab')_2$ fragment it was almost 10 times lower, 17.2 ± 0.8 h.

### *Tumour to normal tissue ratios*

The ratios comparing the radioactivity concentrations in the four different tumours with that of representative normal organs, including liver, kidneys, lung and blood are shown in Figures 2 to 5. The evolution of these ratios showed a marked difference between the intact MAb and the $F(ab')_2$ fragment. For the intact MAb, the mean tumour to blood ratios in mice with tumours Co112, LoVo and 3G7 ranged from 0.8 to 1.05 at 24 h after injection. Surprisingly, these ratios increased only slightly until 96 h after injection when they were 1.0 for LoVo, 1.55 for Co112, and 2.1 for 3G7. In mice with tumour T380, a high tumour to blood ratio of 5.4 was observed as early as 24 h after injection, but again this ratio increased only slightly until 4 days after injection when it reached 8.1. A very similar evolution of the tumour to normal tissue ratios was observed concerning the comparisons with other organs.

In contrast, for the $F(ab')_2$ fragment, tumour to normal

**Table I** Biodistribution of $^{131}I$ labelled intact MAb and its $^{125}I$ labelled $F(ab')_2$ fragment, obtained in mice bearing Co112 xenografts

| | *Hours after injection* | | | | | |
|---|---|---|---|---|---|---|
| | *6* | *12* | *24* | *48* | *96* | *Non-specific localisation at 24 h*[a] |
| *Intact MAb* | | | | | | |
| Tumour | 5.8 ± 0.1[b] | 11.6 ± 3.2 | 12.5 ± 2.4 | 17.3 ± 1.8 | 12.8 ± 3.6 | 6.5 ± 0.7 |
| Liver | 6.4 ± 1.3 | 4.3 ± 0.4 | 3.7 ± 0.4 | 3.3 ± 0.2 | 2.0 ± 0.7 | 3.5 ± 0.3 |
| Kidneys | 5.7 ± 0.7 | 6.2 ± 0.8 | 5.0 ± 0.2 | 4.2 ± 0.3 | 2.7 ± 1.0 | 4.8 ± 0.2 |
| Lung | 7.4 ± 0.6 | 8.6 ± 0.7 | 8.0 ± 1.0 | 6.9 ± 0.8 | 4.1 ± 1.3 | 8.0 ± 0.8 |
| Spleen | 3.1 ± 0.4 | 3.7 ± 0.4 | 3.2 ± 0.2 | 2.6 ± 0.4 | 1.3 ± 0.4 | 2.7 ± 0.3 |
| Muscle | 0.6 ± 0.1 | 0.9 ± 0.1 | 1.3 ± 0.3 | 1.1 ± 0.1 | 1.1 ± 0.3 | 1.1 ± 0.0 |
| Bone | 1.8 ± 0.4 | 1.9 ± 0.3 | 1.8 ± 0.3 | 1.8 ± 0.4 | 2.0 ± 0.4 | 1.7 ± 0.1 |
| Blood | 16.3 ± 2.5 | 17.7 ± 0.9 | 15.2 ± 0.7 | 12.3 ± 0.8 | 8.3 ± 2.8 | 13.3 ± 0.5 |
| *F(ab')₂ fragment* | | | | | | |
| Tumour | 6.4 ± 0.5 | 11.2 ± 3.2 | 9.0 ± 2.0 | 6.5 ± 0.6 | 2.46 ± 0.41 | 3.3 ± 0.5 |
| Liver | 4.3 ± 0.8 | 1.7 ± 0.3 | 0.9 ± 0.1 | 0.2 ± 0.1 | 0.04 ± 0.01 | 0.9 ± 0.0 |
| Kidneys | 5.6 ± 0.7 | 3.3 ± 0.5 | 1.7 ± 0.1 | 0.4 ± 0.1 | 0.07 ± 0.03 | 1.8 ± 0.2 |
| Lung | 5.9 ± 0.5 | 4.1 ± 0.6 | 2.3 ± 0.2 | 0.7 ± 0.1 | 0.12 ± 0.02 | 2.3 ± 0.2 |
| Spleen | 2.6 ± 0.2 | 1.6 ± 0.3 | 0.9 ± 0.1 | 0.2 ± 0.1 | 0.05 ± 0.02 | 0.8 ± 0.1 |
| Muscle | 0.6 ± 0.1 | 0.6 ± 0.1 | 0.5 ± 0.0 | 0.1 ± 0.0 | 0.05 ± 0.01 | 0.4 ± 0.0 |
| Bone | 1.4 ± 0.3 | 1.0 ± 0.1 | 0.6 ± 0.1 | 0.3 ± 0.0 | 0.08 ± 0.01 | 0.6 ± 0.0 |
| Blood | 9.4 ± 1.4 | 5.8 ± 0.6 | 2.9 ± 0.2 | 0.7 ± 0.1 | 0.11 ± 0.04 | 2.7 ± 0.1 |

[a]Non-specific tumour localisation at 24 h was obtained in mice after saturation of tumour CEA by pre-injection of the animals with 1.5 mg unlabelled antibody of identical specificity. [b]Mean percent injected dose per gram tissue ± 1 standard deviation (after correction for physical half-lives of the isotopes) was obtained from three to four mice analysed at different times after injection.

**Table II** Biodistribution of $^{125}$I labelled intact MAb and its $^{131}$I labelled F(ab')$_2$ fragment, obtained in mice bearing LoVo xenografts

| | *Hours after injection* 6 | 12 | 24 | 48 | 96 | *Non-specific localisation at 24 h*[a] |
|---|---|---|---|---|---|---|
| *Intact MAb* | | | | | | |
| Tumour | 6.5 ± 0.3[b] | 11.7 ± 2.1 | 14.2 ± 2.4 | 12.5 ± 1.1 | 10.5 ± 3.3 | 6.3 ± 0.6 |
| Liver | 6.6 ± 2.2 | 4.6 ± 0.4 | 3.7 ± 0.3 | 3.0 ± 0.3 | 2.8 ± 0.6 | 3.3 ± 0.3 |
| Kidneys | 6.9 ± 0.2 | 5.4 ± 0.6 | 5.1 ± 0.3 | 4.0 ± 0.5 | 3.3 ± 0.4 | 5.2 ± 0.6 |
| Lung | 11.2 ± 2.7 | 9.2 ± 1.5 | 8.9 ± 1.3 | 7.2 ± 0.5 | 6.2 ± 1.0 | 7.6 ± 1.0 |
| Spleen | 4.2 ± 0.3 | 4.3 ± 0.9 | 3.4 ± 0.2 | 2.4 ± 0.4 | 2.7 ± 0.7 | 2.8 ± 0.2 |
| Muscle | 0.7 ± 0.2 | 1.0 ± 0.1 | 1.4 ± 0.2 | 1.3 ± 0.2 | 1.2 ± 0.3 | 1.2 ± 0.2 |
| Bone | 2.7 ± 0.0 | 2.1 ± 0.3 | 2.4 ± 0.1 | 2.0 ± 0.1 | 1.7 ± 0.2 | 2.2 ± 0.3 |
| Blood | 22.6 ± 1.3 | 19.0 ± 1.7 | 16.0 ± 1.4 | 13.2 ± 1.4 | 11.5 ± 3.7 | 15.4 ± 1.4 |
| *F(ab')$_2$ fragment* | | | | | | |
| Tumour | 7.4 ± 0.5 | 11.1 ± 2.3 | 9.0 ± 1.0 | 4.6 ± 0.5 | 1.34 ± 0.52 | 2.1 ± 0.3 |
| Liver | 4.6 ± 1.3 | 2.1 ± 0.2 | 0.9 ± 0.1 | 0.3 ± 0.0 | 0.04 ± 0.01 | 0.8 ± 0.1 |
| Kidneys | 8.4 ± 0.6 | 4.1 ± 0.3 | 1.9 ± 0.2 | 0.6 ± 0.1 | 0.07 ± 0.01 | 1.7 ± 0.1 |
| Lung | 9.1 ± 1.8 | 5.4 ± 0.8 | 2.7 ± 0.5 | 0.8 ± 0.0 | 0.15 ± 0.01 | 2.3 ± 0.2 |
| Spleen | 3.4 ± 0.3 | 2.2 ± 0.4 | 0.9 ± 0.1 | 0.2 ± 0.1 | 0.08 ± 0.04 | 0.7 ± 0.1 |
| Muscle | 0.8 ± 0.2 | 0.8 ± 0.1 | 0.5 ± 0.0 | 0.2 ± 0.0 | 0.03 ± 0.01 | 0.4 ± 0.0 |
| Bone | 2.5 ± 0.1 | 1.4 ± 0.2 | 0.9 ± 0.2 | 0.3 ± 0.0 | 0.06 ± 0.01 | 0.8 ± 0.1 |
| Blood | 14.1 ± 0.9 | 7.6 ± 0.7 | 3.1 ± 0.2 | 0.8 ± 0.1 | 0.11 ± 0.05 | 2.9 ± 0.2 |

[a,b]Legends as described in Table I.

**Table III** Biodistribution of $^{125}$I labelled intact MAb and its $^{131}$I labelled F(ab')$_2$ fragment, obtained in mice bearing 3G7 xenografts

| | *Hours after injection* 6 | 12 | 24 | 48 | 96 | *Non-specific localisation at 24 h*[a] |
|---|---|---|---|---|---|---|
| *Intact MAb* | | | | | | |
| Tumour | 7.3 ± 0.7[b] | 11.8 ± 2.8 | 17.9 ± 1.9 | 21.6 ± 2.9 | 17.7 ± 4.3 | 6.2 ± 1.7 |
| Liver | 7.4 ± 1.4 | 4.1 ± 0.7 | 5.0 ± 0.4 | 2.8 ± 0.3 | 2.2 ± 0.2 | 4.4 ± 0.4 |
| Kidneys | 6.8 ± 0.8 | 5.4 ± 0.8 | 4.9 ± 0.3 | 3.7 ± 0.1 | 2.6 ± 0.1 | 6.1 ± 2.3 |
| Lung | 7.5 ± 0.5 | 8.5 ± 1.0 | 9.1 ± 0.2 | 6.2 ± 0.3 | 4.7 ± 0.2 | 7.2 ± 2.3 |
| Spleen | 3.9 ± 0.4 | 3.6 ± 0.6 | 4.4 ± 1.2 | 2.3 ± 0.1 | 1.7 ± 0.1 | 3.8 ± 1.4 |
| Muscle | 0.7 ± 0.1 | 1.0 ± 0.1 | 1.2 ± 0.2 | 1.2 ± 0.0 | 1.1 ± 0.2 | 1.4 ± 0.2 |
| Bone | 2.1 ± 0.9 | 1.9 ± 0.1 | 1.8 ± 0.3 | 2.1 ± 0.3 | 1.5 ± 0.5 | 2.8 ± 1.7 |
| Blood | 22.0 ± 1.5 | 18.7 ± 2.1 | 16.9 ± 1.6 | 11.9 ± 0.7 | 8.3 ± 1.6 | 15.0 ± 2.4 |
| *F(ab')$_2$ fragment* | | | | | | |
| Tumour | 7.2 ± 0.8 | 8.9 ± 1.8 | 11.4 ± 1.1 | 8.8 ± 1.0 | 3.70 ± 1.31 | 2.6 ± 1.2 |
| Liver | 5.6 ± 0.9 | 1.8 ± 0.2 | 1.3 ± 0.1 | 0.3 ± 0.1 | 0.04 ± 0.01 | 1.4 ± 0.5 |
| Kidneys | 8.4 ± 0.8 | 3.7 ± 0.4 | 2.3 ± 0.2 | 0.7 ± 0.1 | 0.12 ± 0.03 | 2.4 ± 0.5 |
| Lung | 8.3 ± 0.8 | 4.5 ± 0.3 | 3.3 ± 0.2 | 1.1 ± 0.1 | 0.22 ± 0.02 | 2.5 ± 0.6 |
| Spleen | 3.5 ± 0.3 | 1.7 ± 0.2 | 1.2 ± 0.2 | 0.3 ± 0.1 | 0.05 ± 0.01 | 1.3 ± 0.6 |
| Muscle | 0.7 ± 0.1 | 0.6 ± 0.0 | 0.5 ± 0.1 | 0.2 ± 0.0 | 0.04 ± 0.01 | 0.5 ± 0.1 |
| Bone | 1.6 ± 0.9 | 1.1 ± 0.0 | 0.7 ± 0.1 | 0.3 ± 0.0 | 0.04 ± 0.01 | 0.9 ± 0.3 |
| Blood | 14.4 ± 1.1 | 6.8 ± 0.6 | 3.6 ± 0.4 | 0.9 ± 0.1 | 0.12 ± 0.05 | 3.3 ± 0.6 |

[a,b]Legends as described in Table I.

**Figure 2** Tumour to normal tissue ratios obtained with $^{125}$I labelled intact chimeric anti-CEA MAb (broken lines) and its $^{131}$I labelled F(ab')$_2$ fragment (full lines), injected simultaneously into nude mice bearing human colon carcinoma xenografts of T380. The ratios shown are tumour to blood (□), tumour to liver (△), tumour to kidneys (■) and tumour to lung (▲) at different times (6, 12, 24, 48, and 96 h) after injection. Vertical lines represent 1 standard deviation calculated from groups of three to four animals.

**Figure 3** Tumour to normal tissue ratios obtained in nude mice bearing human colon carcinoma xenografts of Co112. Legend is as described in Figure 2, except that intact MAb was labelled with $^{131}$I and F(ab')$_2$ with $^{125}$I.

**Figure 4** Tumour to normal tissue ratios obtained in nude mice bearing human colon carcinoma xenografts of LoVo. Legend is as described in Figure 2.

**Figure 5** Tumour to normal tissue ratios obtained in nude mice bearing rat colon carcinoma xenografts 3G7 transfected with human CEA cDNA. Legend is as described in Figure 2.

tissue ratios increased steadily until 4 days after injection (Figures 2 to 5). In mice bearing tumour T380 xenografts, the tumour to blood ratio at 24 h was already 16.6 and increased to 45 at 96 h after injection. This represents an increase of tumour to blood ratios for $F(ab')_2$ compared to the values obtained with the intact MAb in the same animals of 3.1-fold at 24 h and of 5.3-fold at 96 h (Table IV). The tumour to blood ratios for the $F(ab')_2$ fragment in the three other groups of mice bearing tumours Col12, LoVo and 3G7 were 3.1, 2.9 and 3.2 at 24 h, and 24.5, 14.5 and 34.9 at 96 h, respectively. These results obtained with $F(ab')_2$ represent an advantage of tumour to blood ratios over the intact antibody of 3- to 3.8-fold at 24 h and of 14.3- to 16.1-fold at 96 h after injection (Table IV). Note the remarkably low variations of these results that are a consequence of the direct comparison in each animal of intact MAb with the $F(ab')_2$ fragment.

The tumour to blood ratios obtained with intact MAb were compared statistically to those obtained with the $F(ab')_2$: for each group of three to four animals analysed at the five different dissection times, and for all four tumours, the differences for tumour to blood ratios between intact MAb and $F(ab')_2$ were statistically significant (Student *t*-test, 20 analysis). The statistical significance was low for five out of 20 comparisons ($2p<0.05$), while for the 15 other comparisons the difference was highly significant ($2p<0.01$).

### Discussion

The tumour uptake and biodistribution of $F(ab')_2$ fragments as compared to intact monoclonal antibodies has been the subject of several studies, generally with the conclusion that higher tumour to normal tissue ratios can be obtained with this type of fragment (Buchegger *et al.*, 1983; Wahl *et al.*, 1983; Colcher *et al.*, 1983; Herlyn *et al.*, 1983). Fragments of smaller size, such as an Fab or even a single-chain Fv, have been less frequently studied in animal models. While high tumour to normal tissue ratios were obtained with such fragments, the absolute amount localised in the tumour was generally very low (Buchegger *et al.*, 1983; King *et al.*, 1992; Milenic *et al.*, 1991).

Comparisons of intact MAbs with their fragments were generally obtained using different groups of mice, bearing the same, arbitrarily selected human tumour xenograft. In the present study, we made this comparison using four different colon tumour xenografts, taking advantage of the paired labelling method and using kinetic analyses, to gain as much information as possible. The results obtained with different tumour xenografts, with a wide range of antibody uptakes from relatively low to very high percentages of injected dose, should be representative of what is observed with tumours in patients. The kinetic evaluation of the tumour uptake and of the tumour to normal tissue ratios at different times after injection allows one to draw some useful conclusions concerning the $F(ab')_2$ fragment as compared to intact antibody, for both clinical immunoscintigraphy and radioimmunotherapy.

In addition, this direct comparison of intact MAb and $F(ab')_2$ in the same mice with the same tumour by the paired labelling method (Pressman *et al.*, 1957) gives more reliable results since all biological properties of the tumour (and of the mice), such as blood flow, vascular permeability and vascular volume are the same for the intact MAb and for the $F(ab')_2$ fragment.

Recently, we have shown that $F(ab')_2$ fragments from chimeric human-mouse MAbs of different subclasses give different tumour uptakes and different circulating half-lives in nude mice (bearing human colon carcinoma xenografts of T380). We found that the $F(ab')_2$ from the human $IgG_2$ subclass gave the highest tumour uptake and the longest half-life in blood as compared to $F(ab')_2$ fragments from other chimeric antibody subclasses.

In the present study, we used this selected $F(ab')_2$ and compared it with the intact chimeric anti-CEA MAb of human $IgG_2$ subclass from which it was derived. In three out of four tumours, T380, LoVo and Col12, maximal localisation of the $F(ab')_2$ occurred at 12 h post injection and was only slightly lower than the maximal localisation obtained

**Table IV** Increase of tumour to blood ratios obtained with $F(ab')_2$ as compared to those obtained with intact MAb, at different times after injection (mean increase ± 1 SD)

| *Hours after injection* | *Number of mice* | *T380* | *Col12* | *LoVo* | *3G7* |
|---|---|---|---|---|---|
| 6 | 3 | 1.7 ± 0.2[a] | 1.9 ± 0.1 | 1.8 ± 0.2 | 1.5 ± 0.1 |
| 12 | 3-4 | 2.2 ± 0.1 | 2.9 ± 0.2 | 2.4 ± 0.1 | 2.1 ± 0.0 |
| 24 | 4 | 3.1 ± 0.2 | 3.8 ± 0.2 | 3.3 ± 0.3 | 3.0 ± 0.1 |
| 48 | 3-4 | 5.0 ± 0.5 | 6.3 ± 0.6 | 6.2 ± 0.5 | 5.5 ± 0.9 |
| 96 | 3 | 5.3 ± 1.7 | 15.5 ± 2.7 | 14.3 ± 1.7 | 16.1 ± 8.0 |

[a]Increase of tumour to blood ratios was obtained by dividing the tumour to blood ratio for $F(ab')_2$ fragment by the respective ratio observed for intact MAb.

with the intact MAb at 24 or 48 h. In the tumour 3G7, maximal localisation with the F(ab')$_2$ fragment occurred relatively late (24 h) and reached only half of the maximal localisation obtained with the intact MAb, that was measured at a late time after injection (48 h). Thus, it appears that in tumours with rapid uptake of both antibody forms such as T380, the fragment can reach maximal tumour localisations similar to those of the intact MAb. In contrast, in a tumour with very slow antibody uptake such as 3G7, the maximal localisation of the fragment remains below that of the intact MAb, probably because during the slow process of tumour localisation, a large part of the F(ab')$_2$ is excreted.

Concerning tumour to normal tissue ratios, for the three tumours with low antibody uptake (Co112, LoVo, and 3G7), the blood radioactivity concentration of intact antibodies remained similar to that of the tumours, even at later times after injection, while for F(ab')$_2$ the blood radioactivity concentration was already 3-fold lower than in the tumour at 24 h and 6- to 10-fold lower at 48 h after injection. The blood radioactivity concentration is of major concern during radioimmunotherapy (Buchegger *et al.*, 1991; Siegel *et al.*, 1990) since bone marrow irradiation, which is the limiting factor of this therapy, is due in great part to circulating radiolabelled antibodies (Press *et al.*, 1989). It is not possible to directly measure bone marrow irradiation in mice, but we have recently shown by precise measurements in a rat model that mean bone marrow radioactivity is about 29 to 40% of that in the blood after injection of $^{131}$I-labelled intact antibodies or F(ab')$_2$ fragments (Buchegger *et al.*, 1991). In the present study, the observation time for intact antibody was too short to calculate precise integral irradiation doses, but the marked difference in the evolution of tumour to blood ratios confirms and extends earlier observations obtained at selected time points (Buchegger *et al.*, 1983; Wahl *et al.*, 1983; Colcher *et al.*, 1983; Herlyn *et al.*, 1983). Tumour to blood ratios with the F(ab')$_2$ fragment were significantly increased as compared to intact MAb, even at 6 to 24 h after injection, and continued to rise, reaching values 14 to 16 times higher than those obtained with intact MAb at 96 h for the three tumours with low antibody uptake.

In order to obtain similar tumour radiation doses, higher amounts of radiolabelled F(ab')$_2$ than of intact antibodies would have to be injected into mice. Nevertheless, the marked differences in tumour to normal tissue ratios obtained here indicate that in radioimmunotherapy, mice treated with F(ab')$_2$ fragments would be exposed to less whole-body and bone marrow radiation than mice treated with intact antibodies while delivering the same dose to the tumour. These results are in agreement with those observed in a comparative radioimmunotherapy performed in T380 tumour bearing nude mice where for the same tumour dose, 50% more blood radiation dose was calculated for intact antibodies as compared to F(ab')$_2$ (Buchegger *et al.*, 1990).

Elevated tumour to normal tissue ratios obtained with F(ab')$_2$ fragments are equally important for immunoscintigraphy since they allow a better interpretation of tumour images in patients (Bischof-Delaloye *et al.*, 1989). Additionally, the advantages of fragments might also be important for immunoscintigraphy or radioimmunotherapy approaches that use two or three step tumour localisation techniques, such as avidin-biotin-antibody (Pervez *et al.*, 1988) or bispecific antibody-radiohapten (Le Doussal *et al.*, 1989, 1990; Goodwin *et al.*, 1988) or antibody-enzyme-prodrug (Bagshawe, 1990), which all strongly depend on a high tumour to normal tissue ratio of antibody at the time of the second or third injection.

We thank Dr F. Healy and Prof D.J. Buchsbaum for reviewing this manuscript. This product was supported by SANDOZ Pharma Ltd, Basel, Switzerland, by the Swiss Foundation for Scientific Research (grant Nr. 31-31238-91), and by 'Recherche Suisse contre le Cancer'.

### References

AUDETTE, M., BUCHEGGER, F., SCHREYER, M. & MACH, J.P. (1987). Monoclonal antibody against carcinoembryonic antigen (CEA) identifies two new forms of crossreacting antigens of molecular weight 90,000 and 160,000 in normal granulocytes. *Mol. Immunol.*, **24**, 1177–1186.

BAGSHAWE, K.D. (1990). Antibody-directed enzyme/prodrug therapy (adept). *Biochem. Soc. Trans.*, **18**, 750–752.

BISCHOF-DELALOYE, A., DELALOYE, B., BUCHEGGER, F., GILGIEN, W., STUDER, A., CURCHOD, S., GIVEL, J.C., MOSIMANN, F., PETTAVEL, J. & MACH, J.P. (1989). Clinical value of immunoscintigraphy in colorectal carcinoma patients: a prospective study. *J. Nucl. Med.*, **30**, 1646–1656.

BREITZ, H.B., WEIDEN, P.L. & VANDERHEYDEN, J.L. (1992). Clinical experience with rhenium-186-labeled monoclonal antibodies for radioimmunotherapy: results of phase I trials. *J. Nucl. Med.*, **33**, 1099–1112.

BUCHEGGER, F., PELEGRIN, A., HARDMAN, N., HEUSSER, C., LUKAS, J., DOLCI, W. & MACH, J.P. (1992). Different behaviour of mouse-human chimeric antibody F(ab')$_2$ fragments of IgG1, IgG2 and IgG4 sub-class *in vivo*. *Int. J. Cancer*, **50**, 416–422.

BUCHEGGER, F., CHALANDON, Y., PELEGRIN, A., HARDMAN, N. & MACH, J.P. (1991). Bone marrow dosimetry in rats using direct tissue counting after injection of radio-iodinated intact monoclonal antibodies or F(ab')$_2$ fragments. *J. Nucl. Med.*, **32**, 1414–1421.

BUCHEGGER, F., PELEGRIN, A., DELALOYE, B., BISCHOF-DELALOYE, A. & MACH, J.P. (1990). 131-I labeled F(ab')$_2$ fragments are more efficient and less toxic than intact anti-CEA antibodies in radioimmunotherapy of large human colon carcinoma grafted in nude mice. *J. Nucl. Med.*, **31**, 1035–1044.

BUCHEGGER, F., PFISTER, C., FOURNIER, K., PREVEL, F., SCHREYER, M., CARREL, S. & MACH, J.P. (1989). Ablation of human colon carcinoma in nude mice by 131-I-labeled monclonal anti-carcinoembryonic antigen antibody F(ab')$_2$ fragments. *J. Clin. Invest.*, **83**, 1449–1456.

BUCHEGGER, F., SCHREYER, M., CARREL, S. & MACH, J.P. (1984). Monoclonal antibodies identify a CEA crossreacting antigen of 95 kD (NCA-95) distinct in antigenicity and tissue distribution from the previously described NCA of 55 kD. *Int. J. Cancer*, **33**, 643–649.

BUCHEGGER, F., HASKELL, C.M., SCHREYER, M., SCAZZIGA, B.R., RANDIN, S., CARREL, S. & MACH, J.P. (1983). Radiolabeled fragments of monoclonal antibodies against carcinoembryonic antigen for localization of human colon carcinoma grafted into nude mice. *J. Exp. Med.*, **158**, 413–427.

CHEUNG, N.K., LANDMEIER, B., NEELY, J., NELSON, D., ABRAMOWSKI, C., ELLERY, S., ADAMS, R.B. & MIRALDI, F. (1986). Complete tumor ablation with iodine 131-radiolabeled disialoganglioside GD2-specific monoclonal antibody against human neuroblastoma xenografted in nude mice. *J. Natl Cancer Inst.*, **77**, 739–745.

COLCHER, D., ZALUTSKY, M., KAPLAN, W., KUFE, D., AUSTIN, F. & SCHLOM, J. (1983). Radiolocalization of human mammary tumors in athymic mice by a monoclonal antibody. *Cancer Res.*, **43**, 736–742.

DREWINKO, B., ROMSDAHL, M.M., YANG, L.Y., AHEARN, M.J. & TRUJILLO, J.M. (1976). Establishment of a human carcinoembryonic antigen-producing colonadenocarcinoma cell line. *Cancer Res.*, **36**, 467–475.

FOLLI, S., PELEGRIN, A., CHALANDON, Y., YO, X., BUCHEGGER, F., LEJEUNE, F. & MACH, J.P. (1993). Tumor Necrosis Factor can enhance radio-antibody uptake in human colon carcinoma xenografts by selective increase of vascular permeability. *Int. J. Cancer*, **53**, 829–836.

GOODWIN, D.A., MEARES, C.F., MCCALL, M.J., MCTIGUE, M. & CHAOVAPONG, W. (1988). Pre-Targeted immunoscintigraphy of murine tumors with indium-111-labeled bifunctional haptens. *J. Nucl. Med.*, **29**, 226–234.

HAMMARSTROM, S., SHIVELY, J.E., PAXTON, R.J., BEATTY, B.G., LARSON, A., GHOSH, R., BORMER, O., BUCHEGGER, F., MACH, J.P., BURTIN, P., SEGUIN, P., DARBOURET, B., DEGORCE, F., SERTOUR, J., JOLU, J.P., FUKS, A., KALTHOFF, H., SCHMIEGEL, W., ARNDT, R., KLOPPEL, G., VON KLEIST, S., GRUNERT, F., SCHWARZ, K., MATSUOKA, Y., KUROKI, M., WAGENER, C., WEBER, T., YACHI, A., IMAI, K., HISHIKAWA, N. & TSUJISAKI, M. (1989). Antigenic sites in carcinoembryonic antigen. *Cancer Res.*, **49**, 4852–4858.

HARDMAN, N., LEE GILL, L., WINTER, R.F.J., WAGNER, K., HOLLIS, M., BUSINGER, F., AMMATURO, D., BUCHEGGER, F., MACH, J.P. & HEUSSER, C. (1989). Generation of a recombinant human-mouse chimaeric monoclonal antibody directed against human carcinoembryonic antigen. *Int. J. Cancer*, **44**, 424–433.

HERLYN, D., POWE, J., ALAVI, A., MATTIS, J.A., HERLYN, M., ERNST, C., VAUM, R. & KOPROWSKI, H. (1983). Radioimmunodetection of human tumor xenografts by monoclonal antibodies. *Cancer Res.*, **43**, 2731–2735.

KING, D.J., MOUNTAIN, A., ADAIR, J.R., OWENS, R.J., HARVEY, A., WEIR, N., PROUDFOOT, K.A., PHIPPS, A., LAWSON, A., RHIND, S.K., PEDLEY, B., BODEN, J., BODEN, R., BEGENT, R.H.J. & YARRANTON, G.T. (1992). Tumor localization of engineered antibody fragments. *Antibody, Immunoconjugates and Radiopharmaceuticals*, **5**, 159–170.

LAMOYI, E. & NISONOFF, A. (1983). Preparation of $F(ab')_2$ fragments from mouse IgG of various subclasses. *J. Immunol. Meth.*, **56**, 235–243.

LE DOUSSAL, J.M., GRUAZ GUYON, A., MARTIN, M., GAUTHEROT, E., DELAAGE, M. & BARBET, J. (1990). Targeting of indium 111-labeled bivalent hapten to human melanoma mediated by bispecific monoclonal antibody conjugates: imaging of tumors hosted in nude mice (erratum appears in *Cancer Res.*, 1990, **50**, 6115). *Cancer Res.*, **50**, 3445–3452.

LE DOUSSAL, J.M., MARTIN, M., GAUTHEROT, E., DELAAGE, M. & BARBET, J. (1989). *In vitro* and *in vivo* targeting of radiolabeled monovalent and divalent haptens with dual specificity monoclonal antibody conjugates: enhanced divalent hapten affinity for cell-bound antibody conjugate. *J. Nucl. Med.*, **30**, 1358–1366.

MACH, J.P., PELEGRIN, A. & BUCHEGGER, F. (1991). Imaging and therapy with monoclonal antibodies in non-hematopoietic tumors. *Curr. Opin. Immunol.*, **3**, 30–33.

MACH, J.P., BUCHEGGER, F., BISCHOF-DELALOYE, A., CURCHOD, S., STUDER, A., DOUGLAS, P., LEYVRAZ, S., GROB, J.P., MOSIMANN, F., GIVEL, J.C., PETTAVEL, J. & DELALOYE, B. (1988). Progress in diagnostic immunoscintigraphy and first approach to radioimmunotherapy of colon carcinoma. In *Radiolabeled Monoclonal Antibodies for Imaging and Therapy*, Srivasta, S. (ed.), pp. 95–109. Plenum Publishing Corp: New York.

MACH, J.P., CARREL, S., MERENDA, C., SORDAT, B. & CEROTTINI, J.C. (1974). *In vivo* localisation of radiolabelled antibodies to carcinoembryonic antigen in human colon carcinoma grafted into nude mice. *Nature*, **248**, 704–706.

MARTIN, K.W. & HALPERN, S.E. (1984). Carcinoembryonic antigen production, secretion and kinetics in BALB/c mice and a nude mouse-human tumor model. *Cancer Res.*, **44**, 5475–5481.

MILENIC, D.E., YOKOTA, T., FILPULA, D.R., FINKELMAN, M.A.J., DODD, S.W., WOOD, J.F., WHITLOW, M., SNOY, P. & SCHLOM, J. (1991). Construction, binding properties, metabolism, and tumor targeting of a single-chain Fv derived from the pancarcinoma monoclonal antibody cc 49. *Cancer Res.*, **51**, 6363–6371.

PERVEZ, S., PAGANELLI, G., EPENETOS, A.A., MOOI, W.J., EVANS, D.J. & KRAUSZ, T. (1988). Localization of biotinylated monoclonal antibody in nude mice bearing subcutaneous and intraperitoneal human tumour xenografts. *Int. J. Cancer*, **3**, 30–33.

PELEGRIN, A., TERSKIKH, A., HAYOZ, D., CHALANDON, Y., OLSSON, N.O., FOLLI, S., BUCHEGGER, F., KROMER, B., SCHWARZ, K., MARTIN, M., MARTIN, F. & MACH, J.P. (1992). Human carcinoembryonic antigen cDNA expressed in rat carcinoma cells can function as target antigen for tumor localization of antibodies in nude rats and as rejection antigen in syngeneic rats. *Int. J. Cancer*, **52**, 110–119.

PRESS, O.W., EARY, J.F., BADGER, C.C., MARTIN, P.J., APPELBAUM, F.R., LEVY, R., MILLER, R., BROWN, S., NELP, W.B., KROHN, K.A., FISHER, D., DESANTES, K., PORTER, B., KIDD, P., THOMAS, E.D. & BERNSTEIN, I.D. (1989). Treatment of refractory non-Hodgkin's lymphoma with radiolabeled MB-1 (Anti-CD37) antibody. *J. Clin. Onc.*, **7**, 1027–1038.

PRESSMAN, D., DAY, E.D. & BLAU, M. (1957). The use of paired labeling in the determination of tumor-localizing antibodies. *Cancer Res.*, **17**, 845–850.

SENEKOWITSCH, R., REIDEL, G., MOLLENSTADT, S., KRIEGEL, H. & PABST, H.W. (1989). Curative radioimmunotherapy of human breast tumors with 131-I-labeled monoclonal antibodies. *J. Nucl. Med.*, **30**, 531–537.

SIEGEL, J.A., WESSELS, B.W., WATSON, E.E., STABIN, M.G., VRIESENDORP, H.M., BRADLEY, E.W., BADGER, C.C., BRILL, A.B., KWOK, C.S., STICKNEY, D.R., ECKERMAN, K.F., FISCHER, D.R., BUCHSBAUM, D.J. & ORDER, S.E. (1990). Bone marrow dosimetry and toxicity for radioimmunotherapy. *Antibody, Immunoconjugates and Radiopharmaceuticals*, **3**, 213–233.

WAHL, R.L., PARKER, C.W. & PHILPOTT, G.W. (1983). Improved radioimaging and tumor localization with monoclonal $F(ab')_2$. *J. Nucl. Med.*, **24**, 316–325.